# Affinity of PET-MRI Tracers for Hypoxic Cells in Breast Cancer: A Systematic Review

**DOI:** 10.3390/cells13121048

**Published:** 2024-06-17

**Authors:** Ioana-Claudia Costin, Loredana G. Marcu

**Affiliations:** 1 Faculty of Physics, West University of Timisoara, 300223 Timisoara, Romania; ioana.costin96@e-uvt.ro; 2 Bihor County Emergency Clinical Hospital, 410167 Oradea, Romania; 3 Faculty of Informatics & Science, University of Oradea, 410087 Oradea, Romania; 4 UniSA Allied Health & Human Performance, University of South Australia, Adelaide, SA 5001, Australia

**Keywords:** hypoxic cell, molecular imaging, radiotracer, hypoxia biomarker, PET, MRI

## Abstract

Tumour hypoxia is a known microenvironmental culprit for treatment resistance, tumour recurrence and promotion of metastatic spread. Despite the long-known existence of this factor within the tumour milieu, hypoxia is still one of the greatest challenges in cancer management. The transition from invasive and less reliable detection methods to more accurate and non-invasive ways to identify and quantify hypoxia was a long process that eventually led to the promising results showed by functional imaging techniques. Hybrid imaging, such as PET-CT, has the great advantage of combining the structural or anatomical image (offered by CT) with the functional or metabolic one (offered by PET). However, in the context of hypoxia, it is only the PET image taken after appropriate radiotracer administration that would supply hypoxia-specific information. To overcome this limitation, the development of the latest hybrid imaging systems, such as PET-MRI, enables a synergistic approach towards hypoxia imaging, with both methods having the potential to provide functional information on the tumour microenvironment. This study is designed as a systematic review of the literature on the newest developments of PET-MRI for the imaging of hypoxic cells in breast cancer. The analysis includes the affinity of various PET-MRI tracers for hypoxia in this patient group as well as the correlations between PET-specific and MRI-specific parameters, to offer a broader view on the potential for the widespread clinical implementation of this hybrid imaging technique.

## 1. Introduction

### 1.1. The Challenge of Hypoxia in Today’s Oncology

Despite the known existence of tumour hypoxia since the 1950’s, today’s oncology is still faced with a number of challenges to overcoming this tumour microenvironmental factor [1]. Hypoxia occurs due to a number of reasons, including tumour angiogenesis that is supported by an imbalance of angiogenic factors causing abnormal vessel growth and development into a vascular network, but also due to blood vessel compression owing to the high interstitial fluid pressure within tumours [2]. Furthermore, cancer expansion via cellular proliferation inherently increases the distance between certain cells and the bordering blood vessels, rendering these cells hypoxic. Hypoxic cells present a treatment challenge for both radiotherapy and chemotherapy, due to their ability to repair radiation-induced sublethal damage in the absence of oxygen and owing to the difficulty of drugs in reaching hypoxic cells through the tumour’s abnormal vasculature [3]. While chronic or diffusion-limited hypoxia is often managed in clinics through fractionated radiotherapy schedules to allow for reoxygenation, acute or perfusion-limited hypoxia presents spatial as well as temporal challenges due its dynamic nature, which makes both detection and treatment more burdensome [3].

Hypoxia has been shown to be responsible for tumour recurrence, being also a promoter of angiogenesis and distant metastasis in solid tumours, as identified through the functions of hypoxia-inducible factor-1α (HIF-1α) [4,5]. HIF-1α is a protein complex activated in tumours under hypoxic conditions (among others), that has a major impact on the dissemination of cancer cells through its regulatory effect along the metastatic pathway, including the creation of premetastatic niches, epithelial–mesenchymal transition, intravasation into blood vessels and extravasation to distant locations [6]. Furthermore, the activation of the HIF pathway represents a stimulus for blood vessel creation from the existing vasculature, regulating the expression of pro-angiogenic factors, such as the vascular endothelial growth factor (VEGF) [7].

A dangerous combination inducive of treatment resistance is the hypoxia–cancer stem cell (CSC) duo, considering the ability of the hypoxic microenvironment to activate signalling pathways (including the upregulation of HIFs) that promote and maintain the self-renewal property of CSCs [8,9]. While cancer stem cells represent only a small fraction of a tumour’s population, their sturdy properties and indefinite proliferative ability render them highly potent in repopulating the tumour [10]. Experimental findings have confirmed the preference of CSCs to reside in hypoxic microenvironmental niches to preserve their stem-like properties, while also offering them protection from the immune system [11].

**Figure 1:**
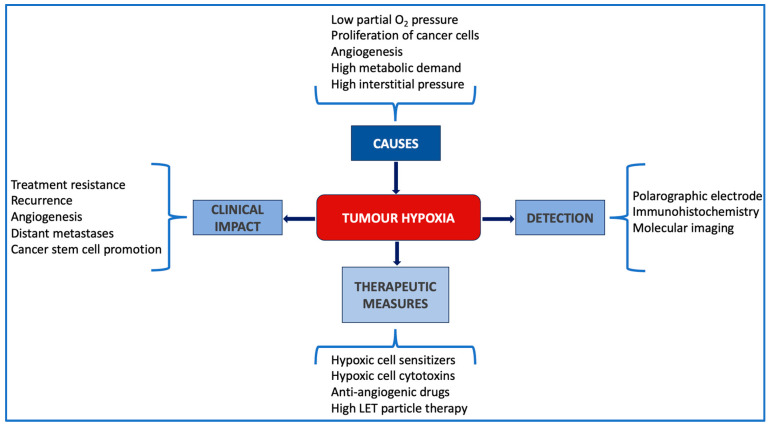
Schematic summary of the main causes of hypoxia, its clinical impact, ways of detection and potential therapeutic measures.

Based on all the above, the identification of hypoxic subvolumes in tumours becomes a key requirement for a successful treatment delivery. Detection of hypoxia has evolved over the decades from invasive methods involving the polarographic electrode to less invasive molecular imaging techniques employing hypoxia-specific tracers [3]. Identification or, ideally, quantification of hypoxia, allows for more personalised treatment planning via various pathways that can include hypoxic cell sensitisers, anti-angiogenic drugs or targeted therapies using radiation with high linear energy transfer and a low oxygen enhancement ratio [12] (Figure 1).

### 1.2. Hypoxia in Breast Cancer

There is clear clinical evidence of the presence of hypoxia in most solid tumours, and breast cancers are no exception [13]. Studies show that among breast cancer patients diagnosed with invasive disease between 25 and 40% display hypoxic subvolumes [13].

The association between breast cancer hypoxia and treatment outcome was evaluated in a large clinical trial with a long follow-up, which included 1178 women after breast-conserving surgery, randomised to receive or not postoperative radiotherapy [14]. Hypoxia was retrospectively analysed using the HIF-1α status and the expression of hypoxic gene signatures. While the benefit of postoperative radiotherapy was shown in both HIF-1α-positive and negative cancers, more recurrences were recorded among patients with HIF-1α-positive primary tumours, this status also being associated with a higher risk of cancer-related deaths (HR = 1.9 [1.2–2.9], *p* = 0.004).

It is acknowledged that different breast cancer subtypes exhibit different responses to anticancer therapies owing to the large number of variables that include genetic as well as tumour-related microenvironmental factors, such as hypoxia. For instance, the overexpression of two key hypoxia-inducible markers—the carbonic anhydrase IX protein (CAIX) and HIF-1α—in early-stage triple-negative breast cancer (TNBC) patients were identified as an unfavourable prognostic factor [15]. A clinical study that evaluated the correlation between hypoxia and tumour response/treatment outcome in a group of 323 TNBC patients showed a strong correlation between the overexpression of CAIX and survival outcomes [16]. CAIX was expressed in 40% of patients, showing significant association with larger tumour size, higher staging and poorer disease-free survival (HR = 2.99 [1.78–5.02], *p* < 0.001), as well as overall survival (HR = 2.56 [1.41–4.65], *p* = 0.002). Based on these findings, the study concluded that overexpression of CAIX is an independent prognostic indicator in triple-negative breast cancer. The clinical validity of these results is confirmed by a recent in vivo pre-clinical study that investigated the capability of a novel carbonic anhydrase inhibitor (para-toluenesulfonamide—PTS) to improve treatment outcome in hypoxic TNBC [17]. The results showed the potential of PTS to downregulate CAIX, HIF-1α and VEGF, while upregulating the expression of apoptosis-related proteins, thus inhibiting breast cancer growth and metastasis in this patient group.

Hormone receptor-positive breast cancers account for most breast cancers and they generally respond well to hormone-based therapy [18]. However, studies evaluating the impact of hypoxia on hormone receptor-positive breast cancers have shown that hypoxia reduces hormone responsiveness in this patient group due to the promotion of oestrogen-independent growth through a significant decrease in oestrogen receptor (ER)-alpha expression levels, rendering the tumour more aggressive [19].

Breast cancers that are positive for the HER2 gene (human epidermal growth factor receptor 2) account for 15–20% of breast cancer patients, and they are associated with poorer clinical outcomes owing to the high risk of distant metastases [20]. Investigating the molecular mechanisms behind the adaptation of HER2+ breast cancer cells to hypoxic conditions, Bashari et al. found a correlation between hypoxia and the anti-apoptotic Mcl-1 (myeloid cell leukemia-1) oncoprotein that is often amplified in breast cancer, showing that the latter confers protection of HER2+ cells to hypoxia [21]. This conclusion followed the observation that the genetic depletion of Mcl-1 lowered both HER2 and HIF-1α levels, hindering the survival of breast cancer cells. Correspondingly, cancer cell death and decreased spheroid formation in HER2+ cells were observed after treatment with EU-5346, a novel small-molecule Mcl-1 inhibitor, which provides support for further clinical developments [21].

In order to identify the main prognostic factors for different subtypes of breast cancer as a function of hypoxia, Li et al. developed a risk model through bioinformatics methods using The Cancer Genome Atlas breast cancer datasets divided into immune-activated and immune-suppressed populations [22]. The latter group was further divided as a function of hypoxia status, using hypoxia-specific genetic signatures. The model showed that among all molecular breast cancer types, HER2+ and ER/PR+ (oestrogen/progesterone receptor-positive) cancers have the highest hypoxia-related risk scores, while EP/PR-negative cancers present with more effective immune pathways, which in turn leads to enhanced response to therapies.

In view of the above, an important step in the management of hypoxic cancers is the identification of hypoxic subvolumes to enable a more personalised approach to treatment. This is partly achievable with functional imaging, a diagnostic field that has greatly developed over the past few decades.

## 2. The Role of Functional Imaging in Detection of Hypoxic Cells in Breast Cancer

Before the development of dual (hybrid) imaging systems, functional imaging such as single-photon emission computed tomography (SPECT) or positron emission tomography (PET) served as a stand-alone tool for the characterisation of a tumour’s molecular properties. In hybrid imaging, a structural or anatomical image (usually provided by CT) is fused with the functional or metabolic image (provided by SPECT or PET) for a more complex depiction of the tumour. The sections below aim to summarise the molecular imaging studies (either hybrid or stand-alone) that have tested various radiotracers for their hypoxia-specific properties and potential clinical use.

### 2.1. SPECT Imaging

SPECT is a functional imaging technique with the potential to identify specific properties within tumour subvolumes, yet the literature reporting on investigations using SPECT for detection of hypoxia is scarce. This might be due to the poorer resolution offered by SPECT images as compared to PET, in situations when the expected information supplied by these molecular imaging techniques must be highly specific and accurate to allow a quantification of the sought parameter (i.e., hypoxia). Among the limited number of studies investigating the role of SPECT in hypoxia imaging, there is a report on the efficiency in detecting hypoxic subvolumes using 99m-Tc labelled with HL91—a hypoxia specific marker—which showed good correlation with 18-F-FGD uptake used in PET and applied to different tumour types, including carcinoma of the breast [23].

Further in vivo studies have shown that high HL91 uptake is associated with poor blood flow, indicating this marker’s affinity towards regions with chronic hypoxia [24]. Biodistribution experiments using 99m-Tc-HL91 in mammary tumour-bearing Wistar rats demonstrated a preferential uptake of the radiotracer in hypoxic regions, correlated with the expression of glucose transporter 1 (GLUT1) which is known to be regulated by hypoxia [25]. While the above results confirmed the potential of HL91 to serve as a hypoxia marker in breast tumours, most investigations involving this tracer were reported before the year 2000, without further follow-up. An explanation for this trend might be found within the new and more promising hypoxia-specific radiotracers developed for PET imaging.

### 2.2. PET Imaging

The increased interest in the quantification of hypoxia in breast cancer over the past few decades is reflected through the various PET radiotracers that have been tested in pre-clinical settings to be further implemented into clinical studies. By far the most common agent used in PET is ^18^F-FDG (F-fluorodeoxyglucose), mainly serving as a staging agent or a differentiator between benign and malignant lesions. This latter observation was not an impediment for some studies to investigate the correlation between the maximum standardised uptake (SUV_max_) of ^18^F-FDG PET-CT and the immunohistochemical expression of HIF-1α in breast cancer patients with invasive ductal cancers [26]. The findings showed a significantly higher SUV_max_ (>6.5) in patients with overexpressed HIF-1α (cut-off value of 2) than in those with lower HIF-1α, concluding that SUV_max_ from ^18^F-FDG PET-CT assessed before radiotherapy can serve as a surrogate marker for hypoxia, thus for progression prediction [26].

Next to ^18^F-FDG, there are hypoxia-specific radiotracers developed for PET-CT imaging which allow for a more direct evaluation/quantification of hypoxic subvolumes, such as ^18^F-MISO (F-fluoromisonidazole), ^18^F-FAZA (F-fluoroazomycinarabinoside), ^18^F-HX4 (F-flortanidazole), ^64^Cu-ATSM (Cu-diacetyl-bis(N4-methylthiosemicarbazone)), etc., examined in a large number of tumours with varying histopathology [27,28,29]. Pre-clinical studies in mice implanted with a human breast cancer cell lines have assessed radiotracer uptake and correlation with hypoxia, through immunohistochemical staining, after administration of either ^18^F-MISO or ^18^F-HX4 [28]. The affinity for hypoxic cells was demonstrated by both radiotracers, their uptake being correlated with the expression of HIF-1α. Furthermore, the tumour-to-normal muscle ratio (T/N) of both ^18^F-MISO and ^18^F-HX4 were positively and significantly correlated with the hypoxic volume (*p* = 0.014 and *p* = 0.009, respectively).

^18^F-MISO has also served as a prognostic indicator in oestrogen-receptor positive breast cancer patients [30]. An observational study including 44 breast cancer patients that underwent ^18^F-FDG as well as ^18^F-MISO PET imaging showed a strong correlation between outcome and ^18^F-MISO uptake measured via tissue-to-blood ratio (TBR). A higher TBR (cut-off of 1.48) was associated with higher levels of VEGF and other angiogenic hypoxic markers. It was concluded that ^18^F-MISO is able to assist with patient stratification identifying those with a baseline risk of early recurrence as well as patients that would benefit from antiangiogenic treatment [30].

A novel hypoxia-specific agent that recently gained interest due to its recent development and synthesis is ^18^F-DiFA (2,2-dihydroxymethyl-3-^18^F-fluoropropyl)-2-nitroimidazole, which has been shown to preferentially accumulate in hypoxic cells through glutathione conjugation following reductive metabolism [31,32]. The biodistribution of the new tracer was comparatively evaluated with ^18^F-MISO in mammary carcinoma-bearing mice, while the presence of hypoxia was confirmed using pimonidazole staining [32]. Biodistribution studies have revealed significantly higher tumour-to-blood and tumour-to-muscle ratios of ^18^F-DiFA as compared to ^18^F-MISO, with a very rapid clearance from other organs, suggesting a better image contrast and a shorter time to scanning with the use of ^18^F-DiFA in hypoxia-specific PET imaging [32]. The efficiency of ^18^F-DiFA as a hypoxia marker has been confirmed by others in human breast cancer-bearing mice treated with eribulin, an inhibitor of microtubule dynamics able to initiate remodelling activity in tumour vasculature [33]. The effects of eribulin on tumour hypoxia were assessed via ^18^F-DiFA, showing a significant reduction in post-treatment tracer uptake, in a dose-dependent manner. The study concluded on the ability of eribulin to induce oxygenation in hypoxic breast cancer cells, also demonstrating the potential of ^18^F-DiFA to serve as an efficient hypoxia marker in PET imaging [33].

Another PET tracer with affinity towards hypoxic cells, ^18^F-FBNA (N-(4-[^18^F]fluoro-benzyl)-2-(2-nitro-1H-imidazol-1-yl)-acet-amide), was newly developed and comparatively assessed against the more established ^18^F-MISO and ^18^F-FAZA in pre-clinical models of triple-negative and oestrogen receptor-positive breast cancer [34]. All three radiotracers showed similar uptake in the hypoxic regions of the two breast cancer models being correlated with elevated levels of HIF-1α expression; additionally, ^18^F-FBNA showed higher tissue clearance compared to ^18^F-MISO.

While most radiotracers used in PET-CT imaging are ^18^F-based, metal-based complexes, such as ^64^Cu-ATSM, have also shown potential in cancer detection, with ^64^Cu offering similar spatial resolution and image quality as the positron energies emitted by ^18^F [35]. The uptake of ^64^Cu-ATSM under hypoxic conditions has been demonstrated in pre-clinical studies using various cell lines. An interesting observation was the cell line dependence shown by ^64^Cu uptake, which might be owing to a different copper metabolism in different cell lines [36]. In breast cancer cells under hypoxic conditions, the uptake and retention of ^64^Cu-ATSM has been confirmed, which warrants further in vivo studies.

### 2.3. The Potential of MRI to Augment Hypoxic Cell Detection

Primarily, MRI has been used as an additional imaging technique for breast cancer diagnosis and staging [37], though the technique also provides information regarding tumour vascularization and subtle soft tissue changes. The MRI-specific quantitative parameters (microvessel density and oxygen-related parameters) provide information about tumour aggressiveness by mapping hypoxia and vascular architecture [38]. In order to detect and quantify vascularization, the perfusion effects and blood oxygen levels are likely to be measure using DCE-MRI (dynamic contrast-enhanced magnetic resonance imaging), which is used to increase the visibility and distinction between tissues [39], or DCE-MRI in combination with other techniques [40].

To validate the potential of MRI to identify tumour-specific properties, the imaging technique is often used in combination with immunohistochemistry in view of correlating MRI-specific quantitative parameters and biomarkers that evaluate tumour cell growth and proliferation (Ki67), metastatic index (HER2), oestrogen receptor (ER), angiogenesis (CD31, VEGFR-2) [41] and also pimonidazole and CAIX staining for the assessment of hypoxia [42]. For instance, the study led by Bennani-Baiti et al. evaluated the potential of MRI to assess tumour hypoxia levels and tumour aggressiveness in breast cancer by correlation with histopathological markers. The results showed a very good co-localisation of hypoxic markers and concluded that aggressive non-luminal tumours presented with higher microvessel density than less aggressive luminal tumours (110.6 mm^−2^ vs. 81 mm^−2^, *p* < 0.01) [38].

While MRI offers valuable tumour-specific information as a sole imaging technique, the main advantage of combining two imaging methods (such as PET and MRI) is to provide a more comprehensive overview of tumour properties, both qualitative and quantitative, by evaluating the associations between PET-specific parameters and MRI-specific factors [42]. The most commonly used MRI and PET parameters according to the literature are presented in Table 1.

## 3. PET-MRI Imaging of Hypoxic Cells in Breast Cancer

### 3.1. Literature Search

Given that the latest hybrid imaging technology employed in oncology is PET-MRI, the current review aimed to analyse the up-to-date scientific reports that investigated the potential of this dual technique to identify hypoxic subregions in breast cancer. In view of this, a systematic search of the scientific literature conducted in the Medline/Pubmed database identified 19 articles using the following keywords: “PET MRI” AND “breast cancer” AND “hypox*”. Due to the limited number of articles obtained, filters related to time period, language and full text were not used, and instead review articles were excluded. In addition, to be consistent with the aim of the study by evaluating hypoxia in breast cancer, other anatomical sites were excluded as well.

**Figure 2:**
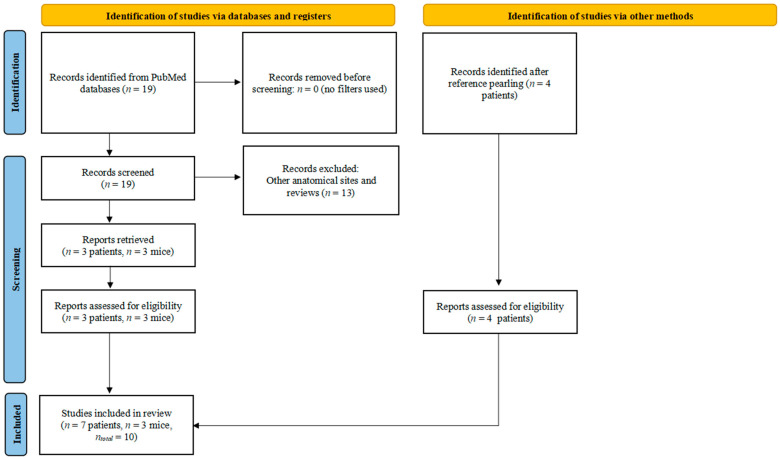
PRISMA diagram for the systematic search of the literature.

Overall, ten articles (seven clinical studies and three pre-clinical studies) were retrieved and evaluated, including those obtained from reference pearling (four clinical studies) (Figure 2). The writing of this systematic review followed the PRISMA guidelines while also adapting to the aims and theme of the research.

### 3.2. Pre-Clinical Studies

Among the evaluated studies, most employed ^18^F-FMISO as a hypoxia-specific PET radiotracer [43,44] (Table 2). This tracer is known to provide spatial information on hypoxia distribution in the tumour volume, which explains its popular use [45]. ^64^Cu-ATSM was also tested as a radiotracer to detect hypoxia and proliferation in triple-negative breast cancer cell lines through the identification of PD-L1 using PET-MRI [46].

The main challenges of hypoxic tumour areas consist of low perfusion, permeability and vascular heterogeneity, leading to certain limitations of ^18^F-MISO imaging [44]. Thus, to supply additional information on the tumour microenvironment, Gertsenshteyn et al. employed DCE-MRI for the evaluation of hypoxic regions in 10 mammary carcinoma-bearing mice, while using EPR (electron paramagnetic resonance) as the standard for partial oxygen pressure (pO_2_) determination [44]. The main aim was the quantification of the accuracy of PET hypoxia imaging as compared to EPR and its correction via DCE-MRI parameters [44]. While the overlap between EPR- and PET-based hypoxia was modest, the MRI-parameter-based algorithm developed within the study was able to correct for PET limitations, offering a more accurate hypoxia map. Since EPR is not a technique to be used in human participants, the study aims to further test the results using immunohistochemistry as a replacement for EPR.

The study conducted by Syed et al. addressed the problem of immunohistochemistry supported by Gertsenshteyn et al. by evaluating 30 BT474 tumour-bearing mice that were treated with trastuzumab (monoclonal antibody against HER2; treated group) or with saline (control group) and imaged with ^18^F-MISO radiotracer [43]. Based on image acquisition and quantitative analysis, K^trans^, ν_e_ and SUV parameters were evaluated using the Kolmogorov–Smirnov (K-S) distance from baseline to endpoint test (day 4 for MRI and day 7 for PET). The K-S distance is a measure of distribution similarity ranging from 0 to 1, where a value closer to 0 represents similar distribution, while a value closer to 1 expresses a different distribution. The correlations obtained by the study were related to the increased vascular heterogeneity on day 4 (K-S distance 0.42) reached by the K^trans^ parameter, increased cellular heterogeneity on day 4 (K-S distance 0.32) obtained by the ν_e_ parameter and decreased hypoxia heterogeneity on day 3 (K-S distance 0.42) and on day 7 (K-S distance 0.46) with narrowing distribution of SUV in the treated group, all under trastuzumab conditions. The control group showed K-S distances of 0.21, 0.22 and 0.28 for vascular heterogeneity, cellular heterogeneity and hypoxia heterogeneity, respectively. Also, for vascular quantification, or cellular or hypoxic heterogeneity from immunohistochemistry data, the tumours were stained with haematoxylin and eosin (HE = measured cellular density), anti-CD31 (measured vessel area), anti-Ki67 (measured proliferating cellular density) or anti-pimonidazoles (measured hypoxia). The significant results were observed in mice treated with trastuzumab, rather than with saline. Under trastuzumab conditions, vascular heterogeneity increased for CD31 in treated groups (1.72%) vs. the control group (0.95%) and hypoxia heterogeneity decreased for pimonidazole in the treated group (0%) vs. the control group (8.05%). The results following the correlations between imaging and immunohistochemistry data showed associations at the vascular heterogeneity level, CD31 vs. K^trans^ (r = 0.33), and hypoxia heterogeneity level, pimonidazole vs. SUV (r = 0.69), while no correlations were found for cell density. Nevertheless, heterogeneity in cellularity under trastuzumab treatment was shown in the ν_e_ wider distribution (K-S distance 0.32), which translates into increased cell death. Furthermore, although a positive correlation was observed between vessel percent area (CD31) and vascular permeability and perfusion (K^trans^) with r = 0.33, immunohistochemistry analysis of vascular heterogeneity on day 4 showed no difference in CD31 between treatment and control. This could be due to the mixed nature of vessel permeability and perfusion of K^trans^. The overall conclusion drawn by the study was that trastuzumab increased cellular and vascular heterogeneity and improved oxygenation across the tumour [43].

The aim of another pre-clinical study involving both murine and human breast cancer cells was to evaluate the role of hypoxia in elevating PD-L1 expression in triple-negative breast cancer, given the limited understanding of the signalling pathways within tumours involving immune checkpoints. The study employed complementary imaging techniques combining bioluminescence (BLI) with PET-MRI using ^64^Cu-avelumab for PET imaging [46]. Following the fusion of the two techniques, an overlap was observed between the increased BLI signal of hypoxia and the high PD-L1 expression detected by PET-MRI, showing a correlation between the two tumour parameters. Additionally, immunohistochemistry analysis identified increased PD-L1 expression in regions near necrosis that were likely hypoxic regions. The conclusion drawn from the study was that PD-L1 is used by cancer cells to inhibit the immune system, and with the influence of hypoxia upon PD-L1 expression, the tumour becomes more aggressive requiring anti-PD-L1 targeted therapy for its management [46].

A monoclonal antibody used to suppress the action of HER2 protein is trastuzumab, which blocks the action of HER2+ by inducing cell cycle arrest, interfering with intracellular signals and inhibiting tumour cell proliferation and migration [47]. In view of this, the study conducted by Sorace et al. observed an improvement in vascularization and intratumoral delivery of drug therapies under trastuzumab treatment in a BT474 mouse model of HER2+ breast cancer [48]. The receiver operating characteristics (ROC) were assessed between a treated group (the group received intraperitoneal injection of trastuzumab) and control group (the group received saline injection) on baseline, day 1 and day 4 for K^trans^ and ν_e_ parameters. The ROC value on day 1 versus day 4 between the treated group and control group for K^trans^ was 0.61 versus 0.86 (*p* = 0.03) and for v_e_ was 0.70 versus 0.91 (*p* = 0.01) [48]. In a subsequent study conducted by the same research group, a reduction in tumour hypoxia in a HER2+ murine model under this agent was confirmed through PET imaging via ^18^F-FMISO, concluding on the potential of trastuzumab to improve tumour perfusion and deliver anti-cancer therapy [49].

### 3.3. Clinical Studies

Characterisation of the tumour microenvironment and the interactions among various factors becomes more feasible with the employment of PET-MRI owing to its multi-parametric combinations. Similar to pre-clinical studies, most clinical reports identified through the literature search used ^18^F-FMISO as a hypoxia-specific PET radiotracer [42,50,51,52,53,54], often administered in combination with ^18^F-FDG to detect metabolically active tumour regions [42,51,55] and/or ^18^F-FLT (F-fluorothymidine) to measure tumour cell proliferation [50,53] (Table 3).

The assessment of cellular hypoxia and heterogeneity within breast cancer patients served as the main goal for the study conducted by Andrzejewski et al. To meet the requirements, both metabolic and hypoxia-specific tracers were administered (^18^F-FDG and ^18^F-FMISO), whereas for the association between hypoxia and distant metastasis, simultaneous contrast MRI and PET images were used. The results highlighted the correlation between ^18^F-FMISO_TBRmean_ and ^18^F-FDG_mean_ with the proliferation factor (r = 0.77 ^18^F-FMISO_TRBmean_ and r = 0.86 ^18^F-FDG_mean_) which indicated an unfavourable prognosis (association with death induced by the disease (r = 0.64 ^18^F-FMISO_TRBmean_ and r = 0.83 ^18^F-FDG_mean_) due to the increased resistance to treatment. In addition, the ratio between the two tracer uptakes and the presence/development of metastasis might suggest that metabolically active tumours with hypoxic regions lead to an increased risk of metastatic spread [42].

In a similar work involving 12 breast cancer patients, Margolis et al. used ^18^F-FDG as a surrogate for tumour aggressiveness and metastatic potential combined with MRI-derived parameters for capillary/vessel permeability (K^trans^ and k_ep_) to find correlations with the Ki67 proliferation factor. Negative correlation was observed between k_ep_ and the occurrence of metastasis (r = −0.59) [56]. Furthermore, a higher value of k_ep_ was indicated in local (0.88 min^−1^) versus distant metastasis (0.45 min^−1^) reflecting increased tumour cellularity. In addition, K^trans^ presented higher values in Ki67+ tumours (0.45 min^−1^) than in Ki67- tumours (0.29 min^−1^), suggesting increased tumour aggressiveness and recurrence due to low plasma flow and vascular permeability. A general conclusion drawn by these studies refers to the clinical value and multiparametric utility of PET-MRI by combining quantitative metabolic and vascular data to guide breast cancer treatment [42,56].

Although, in the study conducted by Margolis et al., K^trans^ showed higher values in Ki67+ tumours, resulting in more aggressive behaviours, the study reported by Carmona-Bozo et al. on 29 ER+ breast cancer patients showed a negative correlation between hypoxic fraction and K^trans^ (r = −0.33) and ν_e_ (r = −0.38), suggesting lower levels of hypoxia with an increase in K^trans^ and ν_e_. Furthermore, they found no correlation between hypoxia parameters (%HF and Ki) and k_ep_ (r = 0.02 and r = 0.08), indicating that tumour hypoxia is more influenced by fluctuations in tumour vascular flow (K^trans^) than capillary permeability (k_ep_) [52]. Another study suggested that k_ep_ would be a more reliable parameter to be used for vessel permeability than K^trans^, because the latter represents a combined measure of blood flow, vessel permeability and capillary surface area [57].

In a later study, Carmona-Bozo et al. further investigated the correlations between morphologic and functional abnormalities of tumour vasculature and the occurrence of tumour hypoxia via ^18^F-FMISO using PET-MRI hybrid imaging [54]. No correlations were reported between DCE-MRI pharmacokinetics (K^trans^, k_ep_, ν_e_, ν_p_) and immunohistochemical indexes (CD31, HIF-1α, CAIX). The study used the influx rate of ^18^F-FMISO as a measure of hypoxia and found an association with hypoxia-induced carbonic anhydrase IX and lower microvessel density and diameter. However, no correlation was observed between HIF-1α and hypoxia-specific PET indexes, with a weak relationship found between HIF-1α and CAIX expression. This result confirms similar findings that in ER+ cancers the overexpression of HIF-1α may be hypoxia-independent and that CAIX may serve as a more robust hypoxia indicator [54,55,58].

To improve tumour oxygenation, a number of studies have reported the administration of drug therapy with bevacizumab as a neoadjuvant treatment [50,53]. Studies have shown that the regulator that can lead to angiogenesis (VEGFR-2) can be blocked by attaching bevacizumab to all forms of the VEGF factor. The study conducted by Lopez-Vega et al. used ^18^F-FLT as a tracer to evaluate tumour proliferation finding a correlation between SUV and Ki67 (ρ = 0.38). The study also used ^18^F-FMISO to detect hypoxia observing a correlation between SUV and VEGFR-2 (ρ = 0.26). The study showed significant reduction in ^18^F-FLT uptake in 52.9% of patients under bevacizumab treatment, observing that tumours with a larger than 25% decrease in hypoxia indicated a reduction in proliferative endothelial cells [53]. Garcia-Foncillas et al. evaluated pathological response using two radiotracers (^18^F-FLT and ^18^F-FMISO) and observed the same reduction in tumour proliferation and perfusion assessed by ^18^F-FLT and MRI under bevacizumab treatment (−26% and −46%, respectively). It was concluded that early changes in tumour hypoxia evaluated via ^18^F-FMISO under bevacizumab treatment can serve as a biomarker of pathological response in breast cancer patients (no correlation between ^18^F-FLT and pathological response) [50].

Ueda et al. evaluated the response to bevacizumab in two groups of breast cancer patients categorised as responders (less glycolysis and hypoxia) and nonresponders (higher glycolysis and severe hypoxia), assessed via combined ^18^F-FDG and ^18^F-FMISO [51]. Tissue oxygen levels were assessed using biomarkers (oxygen saturation SO_2_) and diffuse optical spectroscopic images. Under bevacizumab, oxygen saturation was 68–70% for responders and 62–66% for nonresponders with a baseline of 70.3%. Although studies have shown the benefits of bevacizumab on reducing hypoxia in tumour cells, the study led by Ueda et al. expressed the double personality of this drug: it induces reoxygenation under vasculature remodelling while also leading to severe hypoxia due to vasculature damage. In addition, Lopez-Vega et al. observed a reduction in vascular permeability (decrease in k_ep_ from 260.75 min^−1^ to 158.50 min^−1^ and ν_e_ from 472.5 to 423) owing to inhibition of vascular remodelling induced by angiopoietin-2 (ANGPT2 is a downregulated gene secreted by endothelial cells when vascular remodelling is activated and increases vascular permeability) [53]. A solution for oxygen stabilization suggested by Ueda et al. was the combination of bevacizumab with paclitaxel, which increased the oxygen saturation in responders (70–71%) and nonresponders (69–72%) [51].

## 4. Conclusions

Tumour hypoxia represents one of the key factors leading to cancer recurrence, aggressiveness and metastatic spread in solid tumours, including breast cancer. Therefore, the identification and quantification of hypoxia are important elements of personalised treatment planning, irrespective of the therapeutic method involved. Functional imaging has shown great potential in identifying hypoxic subvolumes within tumours in clinical settings, thus this field sees continuous developments. Hybrid imaging techniques add extra value to image interpretation owing to complementary parameters used by each method in evaluating a specific parameter. In this regard, PET-MRI shows great advantage for hypoxia imaging, since both PET and MRI have the potential to evaluate functional properties of cells from the tumour milieu. The most promising evaluated parameters appear to be K^trans^ and k_ep_ (MRI parameters) due to their ability to supply reliable information on tumour vascular flow, blood flow and vessel permeability. The combination between the above-mentioned parameters and hypoxia-specific parameters (%HF and Ki) would offer an advantage in the evaluation of cellular hypoxia. This overview of the current literature illustrates, through the limited number of studies on breast cancer, the potential of PET-MRI to augment the value of hybrid imaging in the detection of hypoxia. Correlations between PET and MRI parameters, as validated by immunohistochemical data (angiogenesis, hypoxia and cell proliferation-specific parameters), offer new biomarkers that can contribute to a more personalised approach to therapy in this patient group.

## Figures and Tables

**Table 1:** Parameters used in PET-MRI and immunohistochemistry analysis.

Image Indicators	
PET	MRI
K_i_ = tracer influx rate constant	k_ep_ = transfer constant from the interstitial space to the blood plasma
%HF = % of voxels with K_i_ values > 2x standard deviation of mean K_i_ of normoxic tissue	K^trans^ = volume transfer constant between blood plasma and the interstitial space (vascular heterogeneity)
SUV = standardised uptake value (hypoxia heterogeneity)	ν_e_ = extravascular extracellular volume fraction (cellular heterogeneity)
DSS = death induced by the disease	ν_p_ = plasmatic volume fraction
TBR = tumour-to-background ratio calculated as the region of interest SUV normalized to the SUV measured in the patients’ aorta	ADC = apparent diffusion coefficient
**Immunohistochemistry parameters**	
HER2 = human epidermal growth factor receptor 2 = prognoses higher incidence of metastases	
VEGFR-2 = vascular endothelial growth factor receptor-2 = angiogenesis regulator	
CD31 = cluster of differentiation 31 = used to expose the presence of endothelial cells and can evaluate tumour angiogenesis	
HIF-1α = hypoxia-inducible factor 1α = regulates genes involved in angiogenesis, pH regulation, migration and invasion which consists in cancer progression	
CAIX = hypoxia-induced carbonic anhydrase IX	
Ki67 = tumour cell proliferation and growth	
HE = haematoxylin and eosin = identify different types of cells, especially ribosomes and cytoplasmic regions rich in RNA	
Pimonidazole = nitroimidazole with hypoxic selectivity, is reduced in hypoxic environments and can be used as a hypoxia marker	

**Table 2:** Pre-clinical studies that evaluated hypoxia via PET-MRI imaging.

Pre-Clinical Study (Ref.)	MRI Parameter	PET Tracer	Observations
BT747 tumour-bearing mice (Syed et al., 2019 [43])	DCE-MRI	^18^F-FMISO	Under Trastuzumab: K^trans^: longitudinal increase in vascular heterogeneity on day 4 (K-S distance 0.42) ν_e_: longitudinal increase in cellularity heterogeneity on day 4 (K-S distance 0.32) SUV: longitudinal decrease in hypoxia heterogeneity on day 3 (K-S distance 0.42) and on day 7 (K-S distance 0.46) with narrowing distribution of treated SUV
MCa4 mammary carcinoma-bearing mice (Gertsenshteyn et al., 2021 [44])	DCE-MRI	^18^F-FMISO	K^trans^ > 0.25 min^−1^ => higher values => higher perfusion and vascular permeability with high pO_2_ (14 mm Hg > pO_2_ > 60 mm Hg) Lower K^trans^ => hypoxic pO_2_ regions on EPR
4T1 triple-negative breast cancer cell line (Parkins et al., 2023 [46])	DCE-MRI	^64^Cu-avelumab	Mouse and human cells exposed to hypoxia expressed an increase in PD-L1 expression.

**Abbreviations**: ^18^F-FMISO = ^18^F-fluoromisonidazole; K^trans^ = volume transfer constant between blood plasma and the interstitial space; SUV = standardised uptake value; K-S distance = statistic test Kolmogorov–Smirnov distance; ν_e_ = extravascular extracellular volume fraction; PD-L1 = programmed cell death ligand 1.

**Table 3:** Clinical studies using PET-MRI for hypoxia evaluation in breast cancer.

Ref/No. Patients	MRI Parameter	PET Tracer	Results/Validation of Imaging Techniques Against Histopathology
Garcia-Foncillas et al., 2012 [50] 73 patients	-	^18^F-FLT and ^18^F-FMISO	Under bevacizumab: early changes in tumour hypoxia via ^18^F-FMISO serve as biomarker of pathological response
Margolis et al., 2016 [56] 12 patients	K^trans^ and k_ep_	^18^F-FDG	K^trans^ with Ki67−: 0.29 min^−1^ K^trans^ with Ki67+: 0.45 min^−1^ k_ep_ in metastasis (local): 0.88 min^−1^ k_ep_ in metastasis (distant): 0.45 min ^−1^ k_ep_ vs. metastatic burden: r = −0.59
Ueda et al., 2017 [51] 28 patients	-	^18^F-FDG and ^18^F-FMISO	TNBC: FDG vs. FMISO correlated in nonresponders and responders Under bevacizumab (%SO_2_ baseline = 70.3%): responders = 68–70%, nonresponders = 62–66% Under bevacizumab + paclitaxel (%SO_2_ baseline = 70.3%): nonresponders = 69–72%, responders = 70–71%
Andrzejewki et al., 2019 [42] 9 patients with 10 breast cancer lesions	-	^18^F-FDG and ^18^F-FMISO	FMISO_TBR mean_ vs. Ki67: r = 0.77 FDG_mean_ vs. Ki67: r = 0.86 FDG_mean_ vs. DSS: r = 0.83 FDG_TRBmax_/FMISO_TRBmax_ vs. presence/development of metastasis: r = 0.69 FMISO_TRB mean_ vs. DSS: r = 0.64
Carmona-Bozo et al., 2021 [52] 29 patients with 32 breast cancer lesions	K^trans^, k_ep_, ν_e_ and ν_p_	^18^F-FMISO	K_trans_ vs. %HF: r = −0.33, *p* = 0.04 ν_e_ vs. %HF: r = −0.38, *p* = 0.03 k_ep_ vs. %HF: r = 0.02, *p* = 0.90 k_ep_ vs. K_i_: r = 0.08, *p* = 0.65 No correlations between MRI parameters and tumour size %HF vs. pathological size: r = 0.63, *p* < 0.01
Lopez-Vega et al., 2021 [53] 73 patients, efficacy evaluated in 70 patients	Microvessel density, K^trans^ and k_ep_	^18^F-FLT and ^18^F-FMISO	FLT: SUV_max_ vs. Ki67: ρ = 0.38, *p* = 0.001 FMISO: SUV_max_ vs. VEGFR-2: ρ = 0.26, *p* = 0.02 FMISO: SUV_max_ vs. microvessel density: no correlation MRI: k_ep_ vs. FLT SUV_max_: ρ = 0.449, *p* < 0.01 MRI: K^trans^ vs. FLT SUV_max_: ρ = 0.414, *p* < 0.01
Carmona-Bozo et al., 2023 [54] 20 patients with 22 breast cancer lesions	Microvessel density, vessel diameter, K^trans^, k_ep_, ν_e_ and ν_p_	^18^F-FMISO	K_i_ vs. microvessel density: slope = −0.016, r = 0.26, *p* = 0.02 K_i_ vs. vessel diameter: slope = −0.43, r = 0.23, *p* = 0.03 K_i_ vs. CAIX: slope = 1.3 × 10^−4^, r = 0.40, *p* < 0.01 No correlation between MRI parameters and HIF-1α or CAIX

**Abbreviations:** Pathological size = as measured on tumour from patient undergoing primary surgery; TNBC = triple-negative breast cancer; HER2+ = positive human epidermal growth factor recetor-2; [^18^F] FLT = ^18^F-fluorothymidine; [^18^F] FMISO = ^18^F-fluoromisonidazole; [^18^F] FDG = ^18^F-fluorodeoxyglucose; k_ep_ = contrast efflux rate constant; Ki67+ = positive proliferative index; Ki67- = negative proliferative index; %SO_2_ = oxygen saturation percentage; TBR = tumour-to-background ratio; ER = oestrogen receptor; K^trans^ = volume transfer constant between blood plasma and the interstitial space; ν_e_ = extravascular extracellular volume fraction; %HF = percentage hypoxic fraction; K_i_ = influx rate constant; SUV = standardised uptake value; VEGFR-2 = vascular endothelial growth factor receptor-2; CAIX = carbonic anhydrase IX; HIF-1α = hypoxia-inducible factor-1α; DSS = disease-specific death (death induced by the disease); r = Pearson’s correlation coefficient; *p* = Student’s t-distribution *p*-value.

## Data Availability

Data is available from the authors upon request.
